# Microwave ablation with hydrodissection used for the treatment of vascular malformations: effectiveness and safety study

**DOI:** 10.3389/fonc.2024.1146972

**Published:** 2024-06-04

**Authors:** Lu Wang, Man Lu, Min Zhuang, Ying Liang, Shi Shi Wang, Jia Mi Li

**Affiliations:** ^1^ Department of Ultrasound, Sichuan Cancer Hospital, Chengdu, China; ^2^ Department of Clinical Medicine, North Sichuan Medical College, Nanchong, Sichuan, China; ^3^ School of Medical and Life Sciences, Chengdu University of Traditional Chinese Medicine, Chengdu, Sichuan, China

**Keywords:** vascular malformations, hydrodissection, microwave ablation, effectiveness, safety

## Abstract

**Object:**

The aim of the study was to investigate the safety, effectiveness, and peripheral nerve protection in ultrasound-guided microwave ablation (US-guided-MWA) for vascular malformations (VMs) closely related to peripheral nerve.

**Materials and methods:**

From August 2019 to February 2022, 31 patients with 39 VMs received US-guided-MWA. All lesions were confirmed to be closely related to the peripheral nerve by imaging evaluation. Hydrodissection was applied to protect surrounding normal tissue, including peripheral nerves. The patients were followed up at 1day, 2 days, 3 days, 1 week, 1 month, 3 months after operation. Measurements of lesion volume, volume reduction ratio (VRR), sensory and functional abnormalities of adjacent nerves, number of treatments, complication details, personal satisfaction, recurrence, and symptom improvement were recorded.

**Results:**

Among the 39 VMs, the maximum volume is 128.58ml, while the minimum volume is 0.99ml. After a mean follow-up of 13.06 ± 4.83 months, the mean numerical rating scale (NRS) score decreased from 5.13 ± 1.65 to 0.53 ± 0.83 (P<0.0001). The mean mass volume was reduced from 18.34 ± 24.68 ml to 1.35 ± 2.09 ml (P=0.0001). The VRR of all lesions was 92.06%. However, the mean number of treatments was only 1.64 ± 0.87. All patients were satisfied with the technique, with a mean satisfaction score (SC) of 9.23 ± 1.13. There were no motor function abnormalities of the related nerves. 10 patients felt numbness in the ablation area after ablation, and gradually recovered after 1 month.

**Conclusion:**

US-guided-MWA serves as a novel alternative approach for patients with VMs. Preoperative evaluation of the relationship between VMs and peripheral nerves combined with intraoperative hydrodissection is an effective and safe method to prevent nerve injury.

## Introduction

Vascular malformations (VMs) are complex congenital lesions with various clinical manifestations that may affect patients’ lives (1). VMs include capillary malformations, venous malformations, lymphatic malformations, arteriovenous malformations, congenital arteriovenous malformations, and mixed malformations (2, 3). VMs can involve all parts of the body, including dermis, subcutaneous tissue, muscles, bones, and even internal organs. The ones that affect dermis, subcutaneous tissue and skeletal muscle are more common in the head, neck and limbs which lead to a great impact on the appearance (4, 5) and some of them may be close to the peripheral nerves. In addition, VMs are often accompanied by symptoms such as pain, swelling, functional damage and deformity. The ones close to the nerves are more likely to cause pain and dysfunction in the process of growing up (6, 7). Therefore, the impact of VMs, especially those near the nerves, on life quality can be substantial and their treatment should always be taken into account by the clinicians.

Traditionally, the treatment of VMs includes medical appliances, medications, sclerotherapy, surgical resection, and laser ablation. Medications are administered in case of pain, Localized intravascular coagulation (LIC) or consumption coagulopathy (CC). Sclerotherapy is currently the first-line therapy because it is safe and minimally invasive. However, not all the VM will have the same responsiveness to sclerotherapy and multiple treatments are usually required to obtain curative effect. Surgery is not the first choice for VM because it has a great impact on the function and morphology of local tissues, and it may cause severe complications. Laser ablation is limited by availability and expertise and is not suitable for deep or complicated lesions (8–13).

Recently, percutaneous thermal ablation has become popular in clinic as a minimally invasive technique. Among the thermal ablation techniques, microwave ablation (MWA) has a certain therapeutic potential in the treatment of primary and metastatic tumor diseases such as liver disease, lung malignancies, renal tumors et al. due to its larger ablation volumes, shorter ablation durations and higher intra-tumoral temperatures. It is also used for palliative ablation of tumors. In comparison with CT and MRI guided MWA, ultrasound guided MWA (US-guided-MWA) is more convenient and allows real-time guidance with no radiation risk (14–18). Hydrodissection is a standard technique that serves as an effective adjuvant for the thermal ablation, which could be used for separation between the tumor and the surrounding tissues after saline administration into the space between tumor and normal tissues. In that case, thermal damage to the surrounding tissues such as peripheral nerves would be controlled (19–21). Therefore, US-guided-MWA with hydrodissection seem to be an ideal method for minimally invasive tumor therapy like VMs adjacent to nerves.

The purpose of this study is to evaluate the efficacy, safety, and nerve protection of US-guided-MWA with hydrodissection in the treatment of VMs.

## Materials and methods

### Patients

Our retrospective study was approved by the institutional review board and ethical committee of our hospital. Written informed consent was taken from all subjects before the procedures.

From August 2019 to February 2022, 31 patients with a total of 39 VMs referred to our hospital were included in our study. Inclusion criteria were (1) VMs diagnosed as venous malformation, lymphatic malformation, or arteriovenous malformation by fine needle aspiration pathology and imaging; (2) The maximum diameter of the VM was greater than 2cm; (3) The lesions were closely related to the peripheral nerves which can be separated. (4) All the patients had strong desire for treatment due to intolerable symptoms or cosmetic requirements, and voluntarily choose US-guided-MWA. Exclusion criteria were (1) Capillary malformation, congenital arteriovenous fistula; (2) VMs growing around nerves which cannot be separated by hydrodissection; (3) known allergic reaction to CEUS agents; (4) known or suspected cardiopulmonary abnormalities.

All included patients were treated in outpatient department. Information including blood routine, coagulation routine, hepatic and renal function, ECG were collected to exclude inappropriate situations.

**Figure 1:**
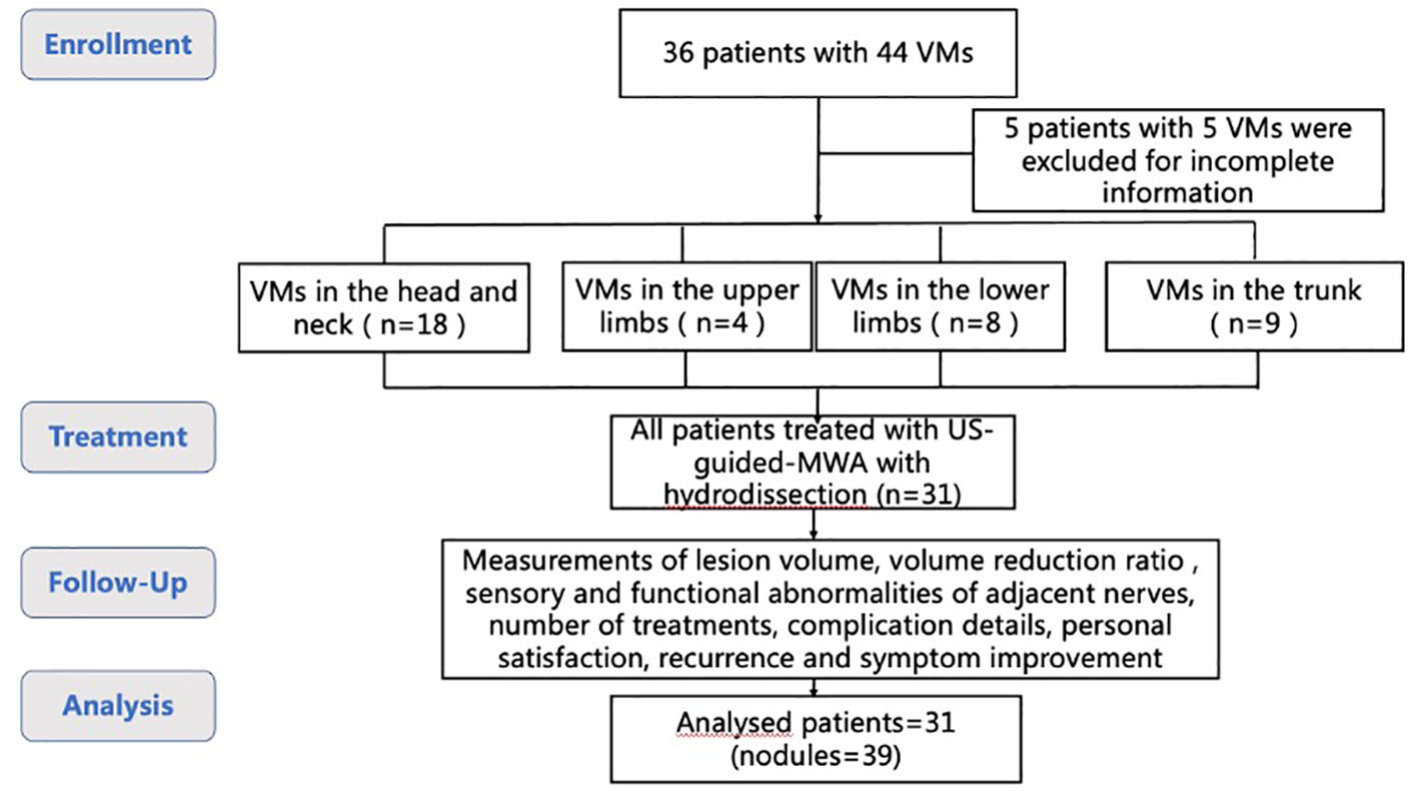
Study protocol flowchart.

A flowchart of the protocol is given in **Figure 1**.

### Image evaluations

**Figure 2:**
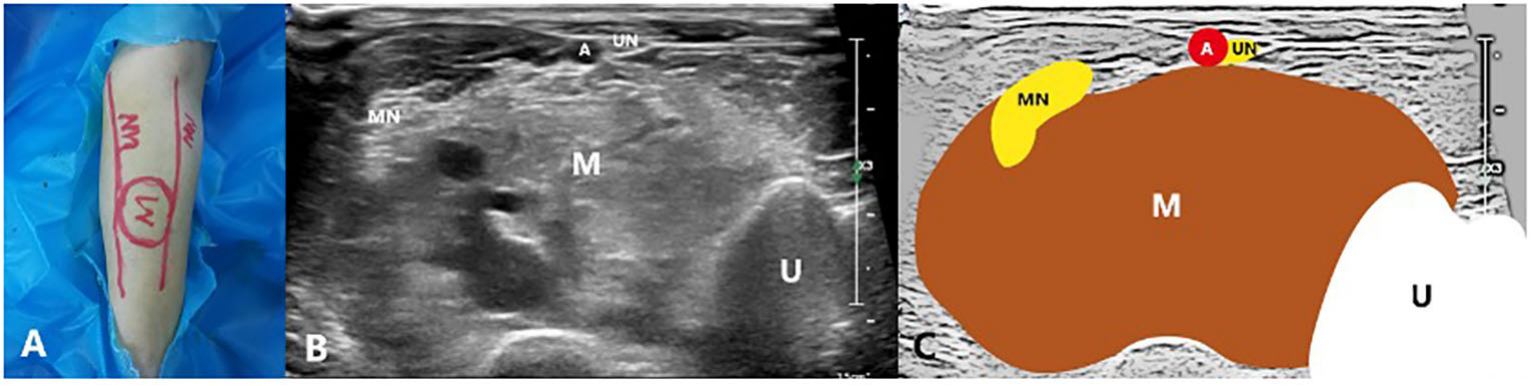
A 27-year-old male with a venous malformation on left arm treated by US-guided-MWA: **(A)** The picture of the neural pathway on the body surface; **(B)** Conventional ultrasound image showing the relationship between the mass and the nerves; **(C)** Schematic diagram of the relationship between the mass and the nerves. M, Mass; MN, Median nerve; UN, Ulnar nerve; U, Ulna; A, Ulnar artery.

Preoperative routine US, CEUS and MRI were used to assess the location, number, extent, demarcation, depth, size of the lesion and its relationship with peripheral nerves. For diffuse vascular malformations, neural pathways need to be marked on the body surface (**Figure 2**). The volume was calculated as follows: Volume=π/6 × length × width × height. The volume reduction ratio (VRR) was calculated as follows: VRR = (initial volume−final volume) ×100%/initial volume.

### Hydrodissection procedure

All operations were performed by one radiologist (M.L.) with over 10 years of interventional experience. A Philips EPIQ7 ultrasound system with a C5-1 (1-5MHz) and a L12-5 (5-12MHz) transducer were used to guide hydrodissection and ablation. During the procedure, the patient was placed in a proper position based on the location of the mass. Routine monitoring including ECG, blood pressure, and intravenous access (iv) were prepared. Before the operation, the safety of ablative pathway was confirmed by US and CEUS.

After disinfection and laying down a sterile towel, anesthesia was performed (**Table 1**). Then, a 22-gauge needle was advanced to the space between the lesion and the surrounding tissue under US guidance. When the needle reached to the targeted space, a 20ml syringe filled with saline was connected to the needle through an extension tube. Saline was injected slowly and continuously while the needle tip slightly adjusted around the VM under the US guidance. The separation was defined successful until the saline zone was at least 1cm wide between the lesion and the peripheral nerve.

**Table 1:** Anesthesia strategy.

The maximum diameter of the VMs	Anesthesia strategy
2-5cm	Local anesthesia
>5cm	Local anesthesia combined with peripheral nerve block

### Ablation procedure

An MWA system (KY-2450A-1; Canyon Medical) with a frequency of 2450 MHz was used to treat the patients.

**Figure 3:**
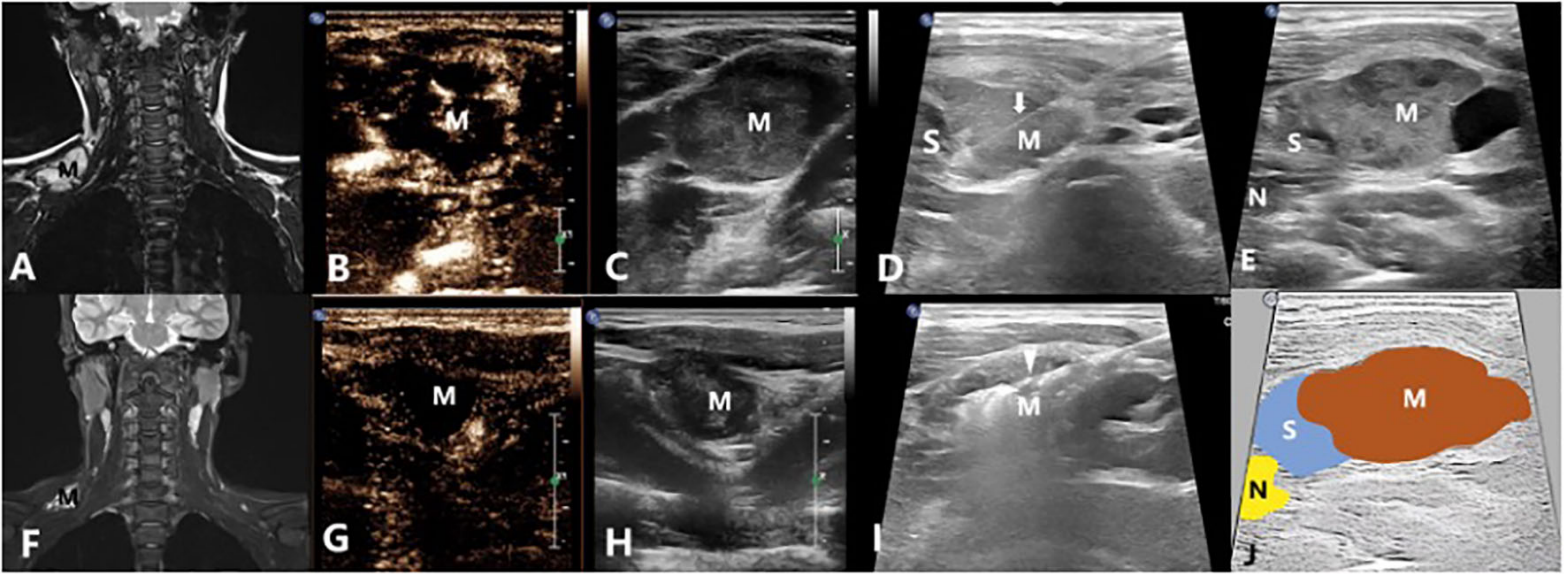
A 14-year-old male with a venous malformation on left shoulder region treated by US-guided-MWA: **(A)** The MRI image of the patient before treatment; **(B, C)** Contrast-enhanced ultrasound of the VM before treatment; **(D)**. Injecting the solution to separate the tumor from normal tissues for protection; **(E)** Conventional ultrasound image showing the relationship between the mass and the nerves; **(F)** The MRI image of the patient 3 months after treatment; **(G, H)** Contrast-enhanced ultrasound of the VM 3 months after treatment; **(I)** US-guided-MWA of the VM; **(J)** Schematic diagram of the relationship between the mass and the nerves. M, Mass; arrow shows the needle which were used to inject the protection solution; arrowhead shows the microwave antenna; N, Cervical plexus; S, The isolation saline.

**Figure 4:**
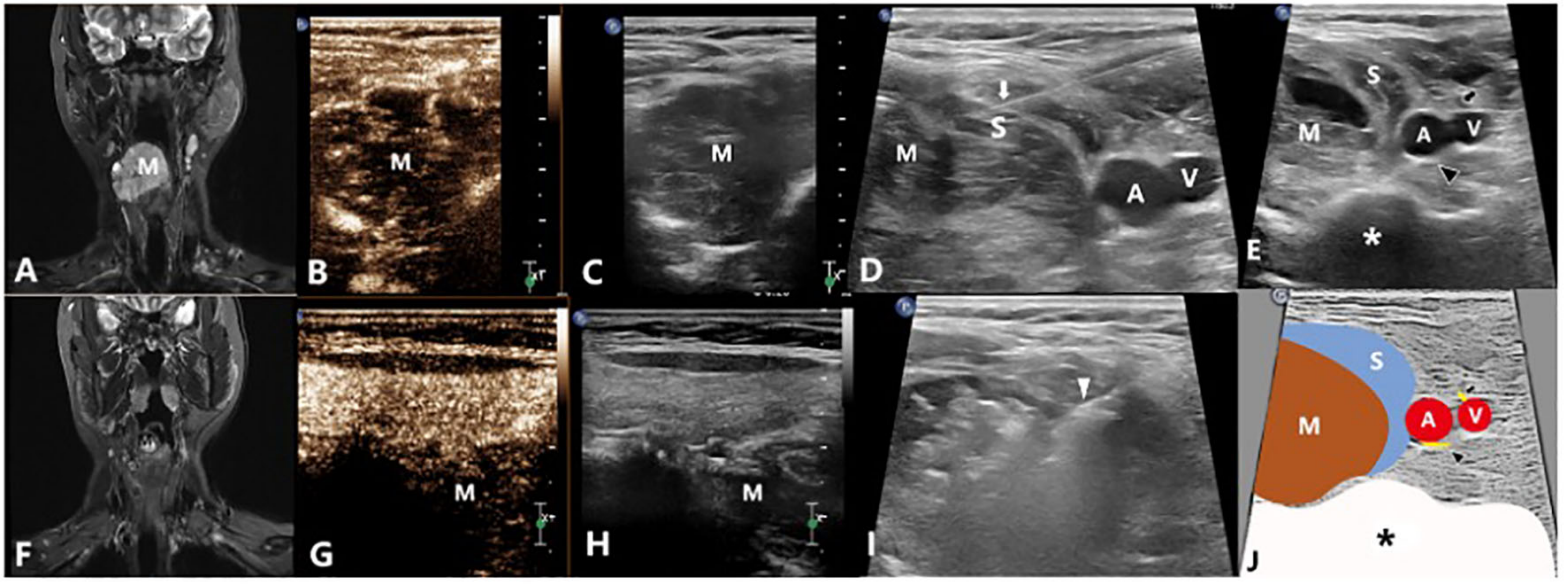
A 34-year-old female with a venous malformation on right neck which grew around the esophagus treated by US-guided-MWA: **(A)** The MRI image of the patient before treatment; **(B, C)** Contrast-enhanced ultrasound of the VM before treatment; **(D)** Injecting the solution to separate the tumor from the normal tissues for protection; **(E)** Conventional ultrasound image showing the relationship between the mass and the nerves; **(F)** The MRI image of the patient 3 months after treatment; **(G, H)** Contrast-enhanced ultrasound of the VM 3 months after treatment; **(I)** US-guided-MWA of the VM; **(J)** Schematic diagram of the relationship between the mass and the nerves. M, Mass; White arrow shows the needle which were used to inject the protection solution; White arrowhead shows the microwave antenna; A: The right common carotid artery; V: The right internal jugular vein; Black arrow shows the vagus nerve; Black arrowhead shows the sympathetic nerve; S, The isolation saline; *, spine.

The microwave antenna was placed at the base of the mass once the protective saline was injected to a satisfactory status. Then, moving-shot ablation technique was performed layer by layer. If the lesion is vascular sinus or venous sinus which is diffuse and dilated, the antenna was placed in the expanded lumen for fixed ablation one by one. The ablation was suspended once the lesion was completely covered by the gas. After the bubbles disappeared 5 minutes later, CEUS was performed to evaluate the ablation. If the ablation area showed no enhancement in arterial phase and venous phase, the ablation was completed (**Figures 3**, **4**). If there was inhomogeneous enhancement or thick tubular enhancement, the lesion needed to be ablated again. If a small amount of residue was confirmed (with a small linear enhancement around), lauromacrogol foam (10ml lauromacrogol injection mixed with 2ml air) was injected into the residue cavity till the gas completely covered the VMs.

After the procedure, all patients were observed for 4 hours on the bed in the observation room of our department to mitigate the risk of adverse events. Symptomatic treatment was given according to the patient’s condition.

### Follow-up

All patients included were followed up at 1 day, 2 days, 3 days, 1 week, 1 month and then once a week after operation. The 1 day, 2 days, 3 days and 1 week follow ups were done by WeChat (an instant communication app). The other follow ups were completed by outpatient service. Measurements of lesion volume, VRR, sensory and functional abnormalities of adjacent nerves, number of treatments, complication details, personal satisfaction, recurrence, and symptom improvement were recorded.

We used numerical rating scale (NRS) (22, 23) to assess the severity of pain. 0 means no pain at all and 10 means the worst pain imaginable.

Based on the Society of Interventional Radiology Clinical Practice Guidelines’ Criteria (16), complications were categorized as minor or major. Imitating the VAS score of pain, we have also developed a satisfaction score (SC). The definition of SC was as follows: satisfaction was evaluated on a scale of 0–10; score 0 represented least satisfied; score 10 was most satisfied.

### Statistical analysis

Two radiologists (W.L and M.Z) acquired and evaluated all the data. Statistical analyses were performed using Medcalc 15.2 and SPSS 22.0 (SPSS Inc.). SC scores and tumor volumes were summarized as mean (X) ± standard deviation (SD). Categorical variables were described as numbers (percentages %). Comparisons between groups were performed with analysis of variance. All statistical tests were two-sided, and the p value<0.05 was considered statistically significant.

## Results

In total, there were 39 VMs from the 31 patients included. 35 of them were venous malformations and 4 were mixed malformations. Among them, 27 were localized lesions (**Figures 3**, **4**) while 12 were diffuse lesions (**Figure 2**). Regarding the location of the lesion, 18 of them were in the head and neck, 4 in the upper limbs, 8 in the lower limbs and 9 in the trunk. The maximum lesion volume was 128.58ml and the minimum volume was 0.99ml. Among the 18 VMs in head and neck, 9 of them were closely related to brachial plexus, 5 were closely related to facial nerves and 4 were closely related to sympathetic nerves. Of those VMs in the upper limbs, 2 had close relationship with ulnar nerve, 2 had close relationship with radial nerve and the other 1 had close relationship with median nerve. For those VMs located in lower limbs, there were 2, 3 and 3 VMs that were closely related to sciatic nerve, peroneal nerve, and tibial nerve respectively. 8 of the 9 VMs located in the trunk were all closely related to intercostal nerve while 1 closely related to brachial plexus (**Table 2**). Each of the 31 patients first received one ablation. Then, 19 patients received no other treatment, 4 received treatment with lauromacrogol sclerotherapy once, 6 received treatment with lauromacrogol sclerotherapy twice, 1 received treatment with lauromacrogol sclerotherapy three times and 1 received the secound ablation. Patients were followed up for a mean of 13.06 ± 4.83 months (median: 13 months; range: 5–21 months) after the last treatment session (**Table 3**).

**Table 2:** Relationship between vascular malformation and nerve.

Location of the VMs	Relationship between vascular malformation and nerve
VMs in the head and neck (n=18)	closely related to brachial plexus (n=9) closely related to facial nerves (n=5) closely related to sympathetic nerves (n=4)
VMs in the upper limbs (n=4)	closely related to ulnar nerve (n=2) closely related to radial nerve (n=2) closely related to median nerve (n=1)
VMs in the lower limbs (n=8)	closely related to sciatic nerve (n=2) closely related to peroneal nerve (n=3) closely related to tibial nerve (n=3)
VMs in the trunk (n=9)	closely related to intercostal nerve (n=8) closely related to brachial plexus (n=1)

**Table 3:** Characteristics of patients.

	31 patients,39 lesions
Sex (F:M)	24:7
Age	39±18(13-70)
Baseline NRS score	5.13±1.65
Location	
Head and neck	18(46.2%)
Trunk	9(23.1%)
Upper extremity	4(10.3%)
Lower extremity	8(20.5%)
Depth of tissue involvement	
Subcutaneous	10(25.6%)
Intramuscular	28(71.8%)
Intra-articular	1(2.6%)
Category	
venous malformations	35(89.7%)
mixed malformations	4(10.3%)

MWA, microwave ablation.

**Figure 5:**
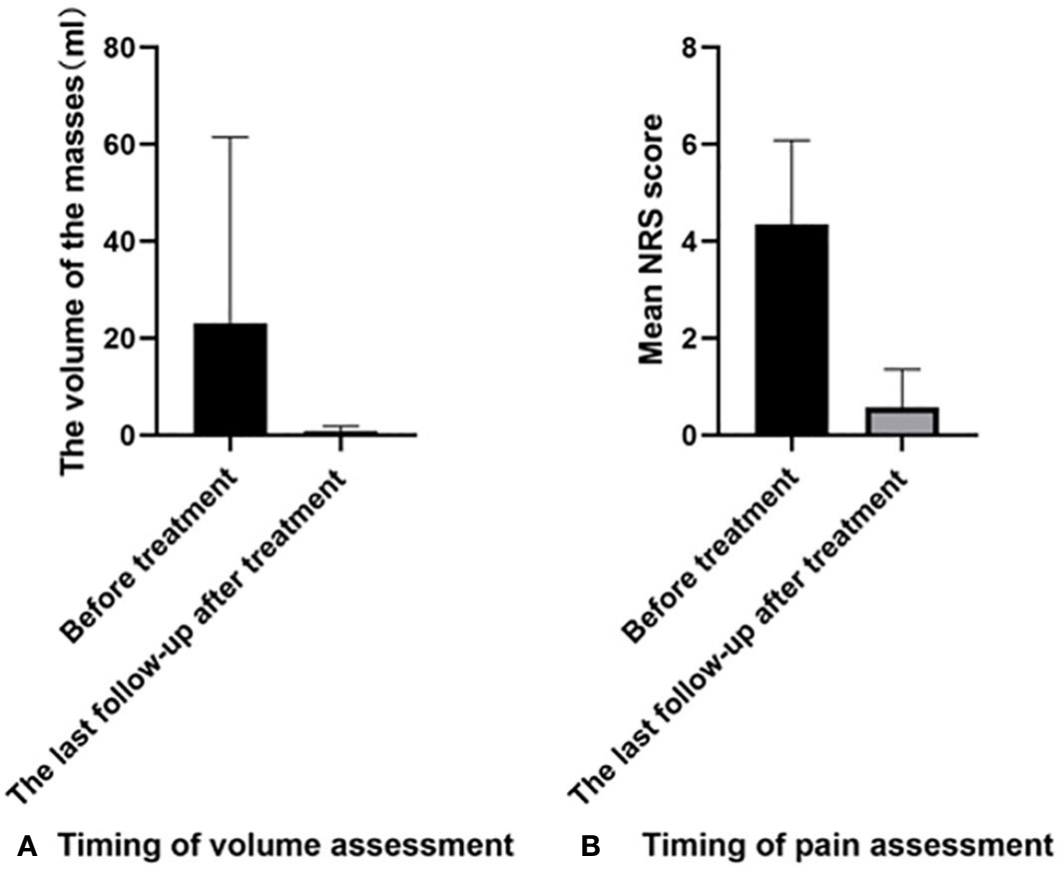
Comparison of different parameters before and after treatment: **(A)** The comparison of volume change; **(B)** The comparison of NRS score. NRS, Numerical rating scale.

One month after the operation, all patients reported a palpable lump in the ablation area, which gradually softened and shrunk during subsequent follow-ups. Much progress has been made in reducing tumor volume, from 18.34 ± 24.68 ml to 1.35 ± 2.09 ml (P=0.0001). The VRR of all lesions was 92.06%. However, the mean number of treatments needed in MWA was only 1.64 ± 0.87. All patients were satisfied with the technique, with a mean satisfaction score (SC) of 9.23 ± 1.13 (**Tables 4**, **5**; **Figure 5**). In terms of pain relief, all patients showed positive responses. The mean NRS score decreased from 5.13 ± 1.65 to before treatment to 0.53 ± 0.83 after treatment (P<0.0001).

**Table 4:** Results of VMs treated by MWA.

Items	
Sessions of treatment	1.64±0.87
SC	9.23±1.13
Complication	7(17.5%)
Positive response in volume reduction	38(97.4%)
Recurrence	3(7.5%)

MWA, microwave ablation.

SC, satisfaction score.

**Table 5:** Changes in volume and VRR of VMs after treatment.

	Tumor volume (median, range, mL)	VRR (median, range, %)
Baseline	18.34±24.68^#^	
The last time follow-up	1.35±2.09^#^	92.06 (47.46-99.89)

VRR, volume reduction ratio.

^#^p<0.001 for comparison between Tumor volume of baseline and the last time follow-up for MWA.

There were no motor function abnormalities with related nerves. 10 patients felt numbness in the ablation area after ablation, and gradually recovered after 1 month. All patients had no intraoperative pain. 80% of patients had mild swelling and pain within 3 days after operation, which gradually disappeared within 1 month.

## Discussion

For VMs, sclerotherapy is considered as the current first-line treatment (1, 11). However, previous studies have shown that it takes time for sclerotherapy to obtain curative effect and not all the VM will have the same responsiveness to sclerotherapy (13, 24–26). This may be because the blood flow of some VMs is at high velocity. The sclerosants flows away quickly. So, the vascular endothelial cells cannot be damaged, resulting in poor effect. In some other cases, venous sinuses are small and have multiple branches. Sclerosing agents cannot fully fill in and destroy the sinus walls. Furthermore, sclerotherapy can be dangerous in some type of VMs. MWA is a new method to the treatment of VMs. Tai et al. used MWA to treat venous malformations with severe localized intravascular coagulopathy. Their efficiency rate was 80% (27).

**Figure 6:**
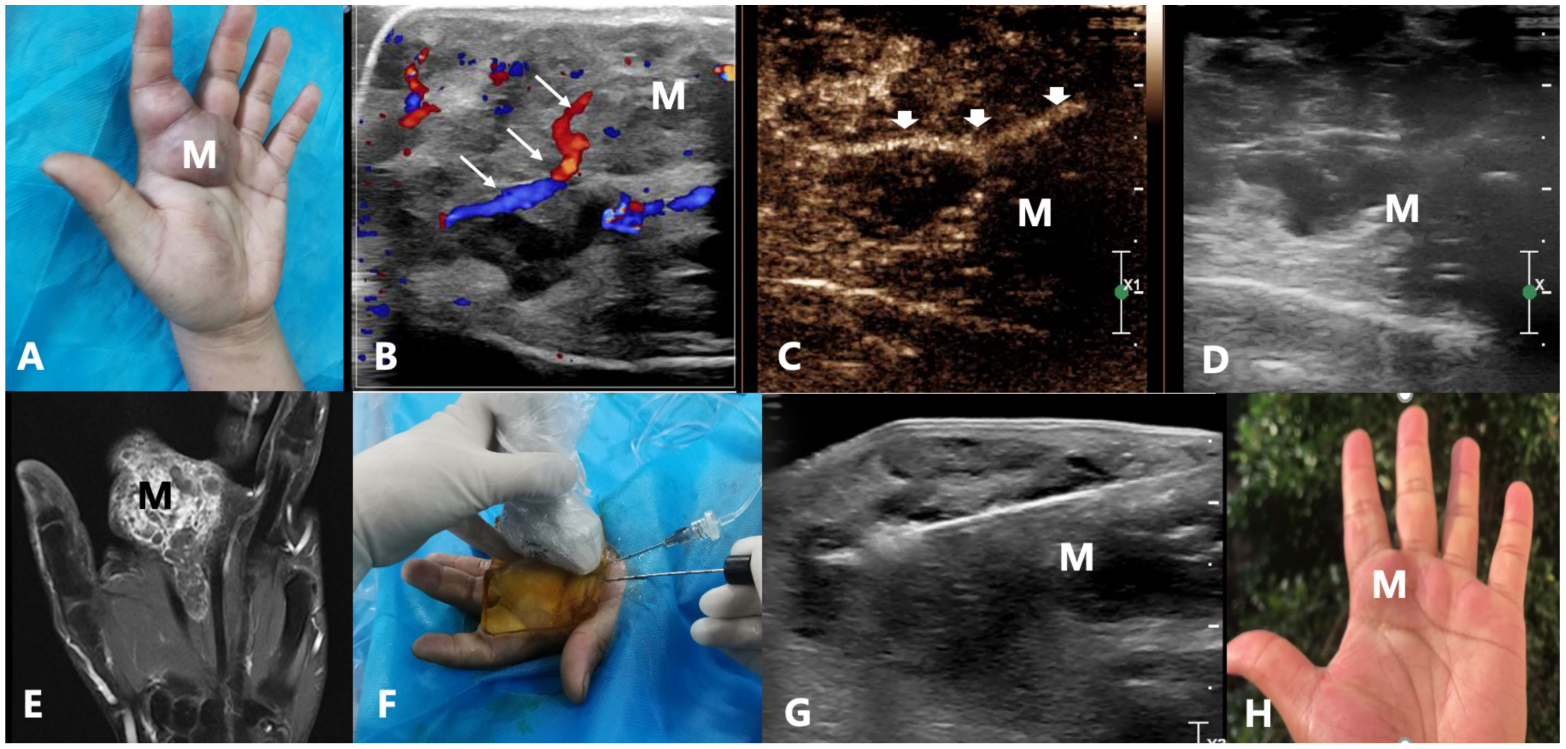
A 35-year-old male with an arteriovenous malformation on the 2nd and 3rd finger spaces of the left palm treated by US-guided-MWA: **(A)** The photo of the patient’ left hand before treatment; **(B)** Color Doppler of the AVM; **(C, D)** Arterial phase of the contrast-enhanced ultrasound of the AVM; **(E)** MRI T1WI imaging of the AVM before treatment; **(F)** The photo of the US-guided-MWA of the AVM; **(G)** The conventional US image of the US-guided-MWA of the AVM; **(H)** The photo of the patient’ left hand 3 months after treatment. M, mass; Long arrow shows the inflow artery on color Doppler image; Shor arrow shows inflow artery on arterial phase of the contrast-enhanced ultrasound image; US, ultrasound; MWA, microwave ablation; AVM, arteriovenous malformation.

In this paper, we used US-guided-MWA to treat VMs. Our results showed that the mean lesion volume was reduced from 18.34 ± 24.68 ml to 1.35 ± 2.09 ml (P=0.0001) and the VRR of all lesions was 92.06%. Otherwise, the positive response in volume reduction was 97.4%. Grieb et al. (24) used polidocanol to treat craniofacial venous malformations. In their study, 90% (18/20) had a post-sclerotherapy improvement in at least one corresponding symptom and expressed satisfaction. In Hou et al.’s study (25), 17.0% of patients had excellent outcomes, 57.4% had good outcomes, 21.3% had fair outcomes, and 4.3% had poor outcomes. Chen et al. (26) found that prolonging the half-life of HA–POL foam sclerotherapy of VMs in the head and neck is safe and effective. Compared with these studies, our results are similar or better. This may be because the heat induced by MWA can permanently damage the vascular endothelium and occlude the blood vessels (**Figure 6**).

In Grieb et al’s (24) study, 56 sessions were administered in 20 patients. The mean session per patient of Hou et al. (25) was 2.1 ± 1.1. The results of Chen et al. (26) showed an overall average of 2.5 treatments per patient. The mean number of treatments of MWA was only 1.64 ± 0.87 which was less than the previous studies. Most of the patients in our hospital are not local, and some are teenagers. They urgently need to complete the treatment and be cured in a short time. The reduction of treatment time can provide convenience to the study and work of patients. Therefore, more patients choose ablation after ultrasound evaluation confirms the safety of ablation.

VMs are commonly located in visible areas which are closely related to peripheral nerves, such as the head and neck. The heat energy of thermal ablation may damage surrounding tissues. Therefore, hydrodissection around the VMs was needed to protect the surrounding tissues. If the injection of protective saline was not satisfactory in the operation, high temperature will lead to nerve damage (27–30). Therefore, it is particularly important to evaluate neural safety preoperatively and to make corresponding plan of safe injection routes and neuroprotective strategy.

In our study, 18 VMs in head and neck were closely related to brachial plexus, facial and/or sympathetic nerves. 4 VMs in upper limbs had close relationship with ulnar nerve, radial or median nerves. 8 VMs in lower limbs were closely associated with sciatic nerve, tibial or peroneal nerves. VMs of trunk were relatively safe, but the intercostal nerve relationship still needs to be evaluated. Most of the cases we included were localized VMs which could be separated from normal tissues by saline. For diffuse VMs, due to the lack of clear boundaries, a nerve routing map on the body surface is needed before operation. During operation, the ablation needle should be mainly positioned in the dilated vascular sinus, while a needle with a drainage tube was placed near the nerve where saline was continuously injected to prevent nerve from thermal injury. As a result, none of our patients had neuromotor dysfunction or limb numbness after operation. Local numbness in the ablation area was observed in 10 patients after operation, which may be caused by may be caused by that the duration of anesthesia is long due to the use of a mixture of lidocaine and ropivacaine for local anesthesia or nerve block. On the other hand, some small nerves (such as cutaneous nerves) in the area may be damaged due to the volume of vascular malformations is large. However, the nerve gradually regenerated and recovered after 1 month.

Other than that, no major complication occurred. Less than half of the patients had minor complications such as pain and mild swelling which showed good response to the symptomatic treatment. This mainly owed to the ultrasound guidance and saline we injected to separate the tumor from normal tissues. In addition, thanks to the use of local anesthesia or nerve blocks, patients experienced no pain during the operation.

Limitations also present in this study. This is a preliminary study on US-guided-MWA used in VMs with a sample size. Our research was limited to venous malformations/arteriovenous malformations and lymphatic malformations, and we excluded VMs growing around nerves. Moreover, we used only two-dimensional ultrasound for preoperative evaluation in this study. If three-dimensional ultrasound is used, the spatial three-dimensional structure, better ablation path plan and intraoperative ablation mode can be provided. In addition, MWA is just one type of ablation methods. Comparisons between different ablation techniques may also be required in the future.

In conclusion, US-guided-MWA serves as a novel alternative approach for patients with VMs. Preoperative evaluation of the relationship between vascular malformations and peripheral nerves combined with intraoperative hydrodissection is an effective and safe method to prevent nerve injury.

## Data availability statement

The raw data supporting the conclusions of this article will be made available by the authors, without undue reservation.

## Ethics statement

The studies involving humans were approved by the institutional review board and ethical committee of Sichuan cancer hospital. The studies were conducted in accordance with the local legislation and institutional requirements. The participants provided their written informed consent to participate in this study.

## Author contributions

LW: Conceptualization, Data curation, Investigation, Methodology, Resources, Software, Validation, Visualization, Writing – original draft. ML: Conceptualization, Funding acquisition, Investigation, Methodology, Project administration, Supervision, Writing – review & editing. MZ: Formal analysis, Resources, Software, Validation, Visualization, Writing – review & editing. YL: Resources, Software, Validation, Visualization, Writing – review & editing. SW: Resources, Software, Writing – review & editing. JL: Resources, Software, Writing – review & editing. All authors contributed to the article and approved the submitted version.
