# Supplementary figures and images for: Microwave ablation with hydrodissection used for the treatment of vascular malformations: effectiveness and safety study
Source: Front Oncol. 2024 Jun 4;14:1146972. doi: 10.3389/fonc.2024.1146972 (PMC11183287; doi:10.3389/fonc.2024.1146972)

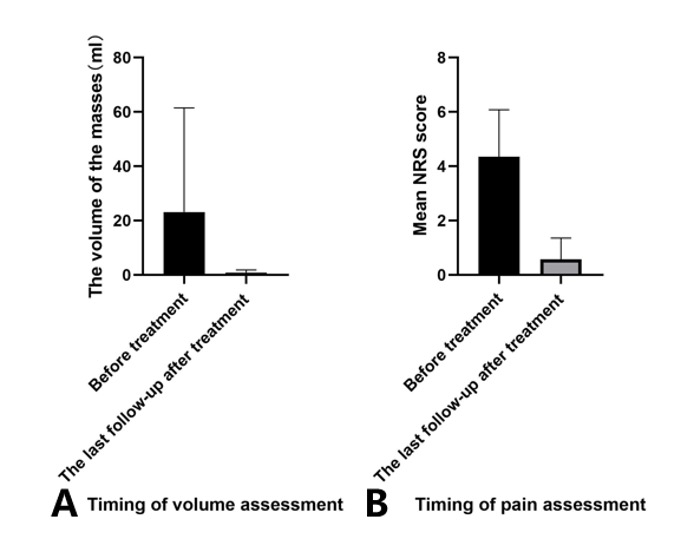

Supplement: Supplementary file 1 [file Image_1.jpeg]
